# Association of plasma exosomes with severity of organ failure and mortality in patients with sepsis

**DOI:** 10.1111/jcmm.15606

**Published:** 2020-07-08

**Authors:** Yunjoo Im, Hongseok Yoo, Jin Young Lee, Junseon Park, Gee Young Suh, Kyeongman Jeon

**Affiliations:** ^1^ Division of Pulmonary and Critical Care Medicine Department of Medicine Samsung Medical Center Sungkyunkwan University School of Medicine Seoul Republic of Korea; ^2^ Department of Critical Care Medicine Samsung Medical Center Sungkyunkwan University School of Medicine Seoul Republic of Korea

**Keywords:** biomarker, exosomes, extracellular vesicles, organ failure, sepsis

## Abstract

Current sepsis biomarkers may be helpful in determining organ failure and evaluating patient clinical course; however, direct molecular biomarkers to predict subsequent organ failure have not yet been discovered. Exosomes, a small population of extracellular vesicles, play an important role in the inflammatory response, coagulation process and cardiac dysfunction in sepsis. Nonetheless, the association of plasma exosome with severity and mortality of sepsis is not well known. Therefore, the overall levels of plasma exosome in sepsis patients were assessed and whether exosome levels were associated with organ failure and mortality was evaluated in the present study. Plasma level of exosomes was measured by ELISA. Among 220 patients with sepsis, 145 (66%) patients were diagnosed with septic shock. A trend of increased exosome levels in control, sepsis and septic shock groups was observed (204 µg/mL vs 525 µg/mL vs 802 µg/mL, *P* < 0.001). A positive linear relationship was observed between overall exosome levels and Sequential Organ Failure Assessment (SOFA) score in the study cohorts (*r* value = 0.47). When patients were divided into two groups according to best cut‐off level, a statistical difference in 28‐ and 90‐day mortality between patients with high and low plasma exosomes was observed. Elevated levels of plasma exosomes were associated with severity of organ failure and predictive of mortality in critically ill patients with sepsis.

## 1 INTRODUCTION

Sepsis is a dysregulated inflammatory response syndrome that leads to life‐threatening organ dysfunction.^1^ The current definition of sepsis identifies a heterogeneous population of individuals with diverse patterns of immune response, organ dysfunction and clinical outcomes.^1^ Current sepsis biomarkers may be helpful in determining organ failure and evaluating the patient's clinical course; however, the use of a single biomarker is insufficient to reflect the complexity of the sepsis pathophysiology,^2^ which is multifactorial and rapidly changing.^3^ Therefore, molecular analysis of sepsis including cytokines and circulating mediators is essential to understand the complexity of cell‐to‐cell communication in multiple organs,^3^, ^4^ which is a decisive response to stressors.

Exosomes are a small population (size range, 30‐100 nm) of extracellular vesicles, formed from the plasma membrane by vesicle budding or intracellular multivesicular bodies by exocytosis^5^ and have transmembrane proteins known as tetraspanins (CD9, CD63, CD37, CD81).^6^ Exosomes have unique features called plasma membrane ligands,^7^ and exosomal cargo that contains proteins, oligonucleotides including DNA, messenger RNA, microRNAs and metabolites such as lipids and glycans.^8^ The circulating exosomes remain stable, and the exosomal cargo is not digested for a long time, which may contribute to cell‐to‐cell communications.^9^, ^10^

Recently, exosomes have been extensively studied as inflammatory markers in several diseases including cancers and cardiovascular diseases.^7^, ^11^ Exosomes play an important role in the inflammatory response,^12^ coagulation process^13^ and cardiac dysfunction,^14^ which all contribute to the multiple pathophysiology of organ dysfunction in sepsis. Moreover, inflammatory cytokines and chemokines, which encapsulated in exosomes including IL‐1β, IL‐2, IL‐6, IL‐12, IL‐15, IL‐17, TNF‐α and IFN‐γ,^15^ so‐called pro‐inflammatory cytokines that promote the synthesis of secondary mediators and migration of inflammatory cells^16^ and lead to collateral tissue damage,^17^ were shown to be associated with the pathophysiology of sepsis.^18^, ^19^ Furthermore, in a study by Chang et al,^20^ adipose‐derived mesenchymal stem cell‐derived exosomes from healthy human reduced systemic inflammation and improved mortality in rat models of sepsis demonstrating that exosomes may have impact on the inflammatory processes of sepsis. In the same manner, immature dendritic cell‐derived exosomes were shown to contain milk fat globule epidermal growth factor VIII which attenuated systemic inflammation by enhancing apoptotic cell clearance.^21^ In addition to their association with inflammatory processes, exosomes are implicated in organ dysfunction in sepsis. In this light, the association between exosome and sepsis and its utility as a biomarker for diagnosis and prognosis has been a particular area of interest. Therefore, the overall levels of plasma exosome in sepsis patients were assessed and whether exosome levels were associated with organ failure and mortality was evaluated in the present study.

## 2 METHODS

This is a prospective, observational study using the Samsung Medical Center Registry of Critical Illness (SMC RoCI), which is an ongoing single centre prospective registry of Samsung Medical Center (1989‐bed, university affiliated, tertiary referral hospital in Seoul, South Korea) initiated in April 2014 for the purpose of establishing a human sample repository and developing new biological markers for critical illness.^22^ Patients or their legally authorized representatives were informed of the purpose of the registry, acquisition of clinical data, obtainment of blood samples and future publication of collected data prior to enrolment. Informed consent was obtained from all patients or their legal representatives. The study was approved by the institutional review board of Samsung Medical Center.

### 2.1 Study population

Critically ill adult patients (≥19 years of age) admitted to the medical intensive care unit (ICU) of Samsung Medical Center were considered eligible for inclusion in the registry. Exclusion criteria were as follows: (a) cognitive impairment, (b) inability to provide informed consent (or lack of an appropriate legal representative to do so), (c) ICU admission for simple procedure or postsurgical care, (d) transfer from other hospitals, (e) end‐of‐life decision or admission to facilitate comfort care, (f) haemoglobin <8 g/dL upon admission or persistent bleeding and (g) discharge within 24 hours of admission to the ICU. Screening and enrolment were completed within 24 hours of ICU admission. A total of 220 patients registered between April 2014 and January 2019 were included in the analysis. In addition, a total of 20 healthy volunteers (≥19 years of age) donated 5 mL of blood samples for research purposes. Written informed consent was obtained from all healthy volunteers.

### 2.2 Clinical data collection

A trained study coordinator used hospital records for each patient to prepare a standardized case report form. Clinical data consisting of patient demographics, reason for ICU admission, severity of illness scoring and laboratory data (PaO_2_/FiO_2_, lactic acid, C‐reactive protein and procalcitonin) were obtained at the time of enrolment. Illness severity was assessed using the Acute Physiology and Chronic Health Evaluation II (APACHE II),^23^ Simplified Acute Physiology Score 3 (SAPS 3)^24^ and Sequential Organ Failure Assessment (SOFA) scores.^25^ The primary outcome was 28‐day mortality, and secondary outcomes were in‐hospital mortality. Sepsis was defined according to the third International Consensus Definitions for Sepsis and Septic Shock (Sepsis‐3).^1^ Since enrolment for the registry began in April 2014, patients enrolled before release of the new definition were reclassified.

### 2.3 Measurement of plasma exosomes

In addition to clinical data, 19 mL of whole blood was drawn from each patient within 48 hours of study enrolment and healthy volunteers. To isolate plasma from whole blood, whole blood was centrifuged at 480 × *g* for 10 minutes at 4°C. Several plasma aliquots from each study participant were isolated and frozen at −80°C until further analysis.

ExoQuick exosome precipitation solution (System Biosciences, Palo Alto, CA, USA) was used to precipitate plasma exosomes. Briefly, plasma was thawed on ice and centrifuged at 1500 *g* for 10 minutes at 4°C. The supernatant was transferred to a new tube, and 2 µL of thrombin (System Bioscience, TMEXO‐1) was added to 250 µL of plasma and incubated for 5 minutes at room temperature to remove fibrinogen. The plasma was then centrifuged at 10 000 rpm for 5 minutes, and the supernatant was collected. The plasma was then incubated with ExoQuick™ for 30 minutes at 4‐5°C. The ExoQuick™/plasma sample was then centrifuged twice at 1500 *g* for 30 and 5 minutes, respectively, in order to remove the supernatant. The pellet was resuspended in 200 µL of PBS. Then, the precipitated exosomes were used immediately.

To assess the size and morphology of exosomes, we performed transmission electron microscopy (TEM) at room temperature after the isolation of exosomes (80 kV, Phillip CM120; Figure S1). The concentration of exosomal proteins was assessed using the PierceTM BCA Protein Assay Kit (Thermo Fisher Scientific, Waltham, MA, USA; 23225) according to the manufacturer's instructions. Samples were separated by 12% or 5% SDS‐PAGE onto nitrocellulose membranes (Thermo Fisher Scientific; 88018). SDS‐PAGE was performed to investigate the presence of common exosome markers (anti‐CD63: Invitrogen, Carlsbad, CA, USA, 10628D, 1:1500; anti‐CD9: Santa Cruz Biotechnology, Dallas, TX, USA, sc‐13118, 1:500). We performed Western blot in a healthy control, two of sepsis patients and two of septic shock patients collected in 2014 and 2019, respectively, to demonstrate the stability of the samples, considering the relatively long storage duration (Figure S2).

To detect and quantify exosomes in plasma, a commercially available ELISA kit (measurement range: 100‐1000 µg), which consists of plates pre‐coated with CD9, a proprietary pan‐exosome antibody, was used according to the manufacturer's protocol (Novusbio, Littleton, CO, USA).

### 2.4 Statistical analysis

Data are presented as numbers (percentages) for categorical variables, and as median and interquartile range (IQR, 25th‐75th percentiles) for continuous variables. Categorical variables were compared using the chi‐square test or Fisher's exact test, and continuous variables were compared using the Mann‐Whitney *U* test. Differences in plasma exosome levels in control, sepsis and septic shock groups were assessed with the Kruskal‐Wallis test.

Associations between exosomes and severity of organ failure, as measured by SOFA score, were assessed using the linear regression. We performed receiver operating characteristic (ROC) analysis to determine the predictive value of exosome level as a prognostic predictor of disease severity and calculated the optimal cut‐off values of exosome level by Youden's index to analyse the relationship between exosome level and disease severity in our cohort. The optimal cut‐off of plasma exosome levels for 28‐day mortality prediction was assessed using Youden's index.^26^ Patients were reclassified into two groups of high and low exosome levels based on the optimal cut‐off level, and initial diagnosis, clinical status, severity of illness and 28‐day mortality were compared between the groups. Kaplan‐Meier equation was used to determine the 90‐day mortality curves according to plasma exosome levels, which were then compared using the log‐rank test.

All tests were two‐sided, and a *P*‐value <0.05 was considered statistically significant. All statistical analyses were performed using R version 3.5.3 (R Foundation for Statistical Computing, http://www.r‐project.org).

## 3 RESULTS

### 3.1 Baseline characteristics

Baseline characteristics of all subjects are presented in Table 1. The median age of patients was 67 years (IQR, 55‐74 years), and 68% were male. During the study period, 145 (67%) patients presented with septic shock. The median Charlson comorbidity index of the patients was 2 points (1‐3 points). A total of 91 (42%) patients required mechanical ventilation, and 166 (76%) patients needed vasopressor support. Significant differences were not observed in age, sex or comorbidities, in 28‐day mortality and 90‐day mortality between sepsis and septic shock patients (Table 1).

**TABLE 1:** Characteristics of sepsis patients based on plasma exosome levels

	Total (n = 220)	Sepsis (n = 75)	Septic shock (n = 145)	*P*‐value
Age, y	67 (55‐74)	65 (52‐76)	67 (58‐73)	.866
Sex, male	150 (68)	55 (73)	95 (66)	.286
BMI, kg/m^2^	22.8 (20.2‐25.6)	22.6 (19.6‐25.0)	23.0 (20.4‐25.7)	.168
Comorbidity				
Diabetes mellitus	72 (33)	20 (27)	52 (36)	.177
Coronary heart disease	11 (5)	3 (4)	8 (6)	.753
Chronic kidney disease	16 (7)	6 (8)	10 (7)	.788
Solid tumour	73 (33)	22 (29)	51 (35)	.451
Haematologic malignancy	32 (15)	13 (17)	19 (13)	.424
Charlson comorbidity index	2 (1‐3)	2 (1‐3)	2 (1‐3)	.624
Clinical status on ICU admission				
Need for mechanical ventilation	91 (42)	26 (35)	65 (45)	.192
Need for vasopressor support	166 (76)	21 (28)	145 (100)	<.001
Laboratory findings				
PaO_2_/FiO_2_	193 (127‐305)	192 (132‐287)	194 (124‐306)	.615
Lactic acid (mmol/L, n = 220)	2.85 (1.89‐4.23)	1.69 (1.24‐1.92)	3.58 (2.64‐5.17)	<.001
CRP (mg/dL, n = 218)	13.07 (5.76‐24.17)	13.33 (5.28‐24.75)	12.93 (5.83‐24.04)	.991
PCT (ng/mL, n = 184)	5.12 (0.86‐21.51)	1.00 (0.27‐5.50)	7.92 (1.46‐34.17)	<.001
Exosome (μg/mL, n = 220)	795 (558‐826)	525 (499‐575)	802 (783‐839)	<.001
Severity of illness				
SAPS 3 score	54 (47‐62)	49 (40‐57)	57 (51‐65)	<.001
APACHE II score	24 (19‐30)	23 (17‐28)	24 (20‐30)	.021
Initial SOFA score	9 (6‐11)	6 (4‐9)	10 (8‐12)	<.001
Mortality				
28‐d mortality	39 (18)	12 (16)	27 (19)	.712
In‐hospital mortality	53 (24)	17 (23)	36 (25)	.868
90‐d mortality	73 (33)	23 (31)	50 (35)	.651

Note

Data are presented as median (interquartile range) or number (%).

Abbreviations: APACHE, Acute Physiology and Chronic Health Evaluation; BMI, body mass index; CRP, C‐reactive protein; ICU, intensive care unit; PCT, procalcitonin; SAPS, Simplified Acute Physiology Score; SOFA, Sequential Organ Failure Assessment.

**FIGURE 1:**
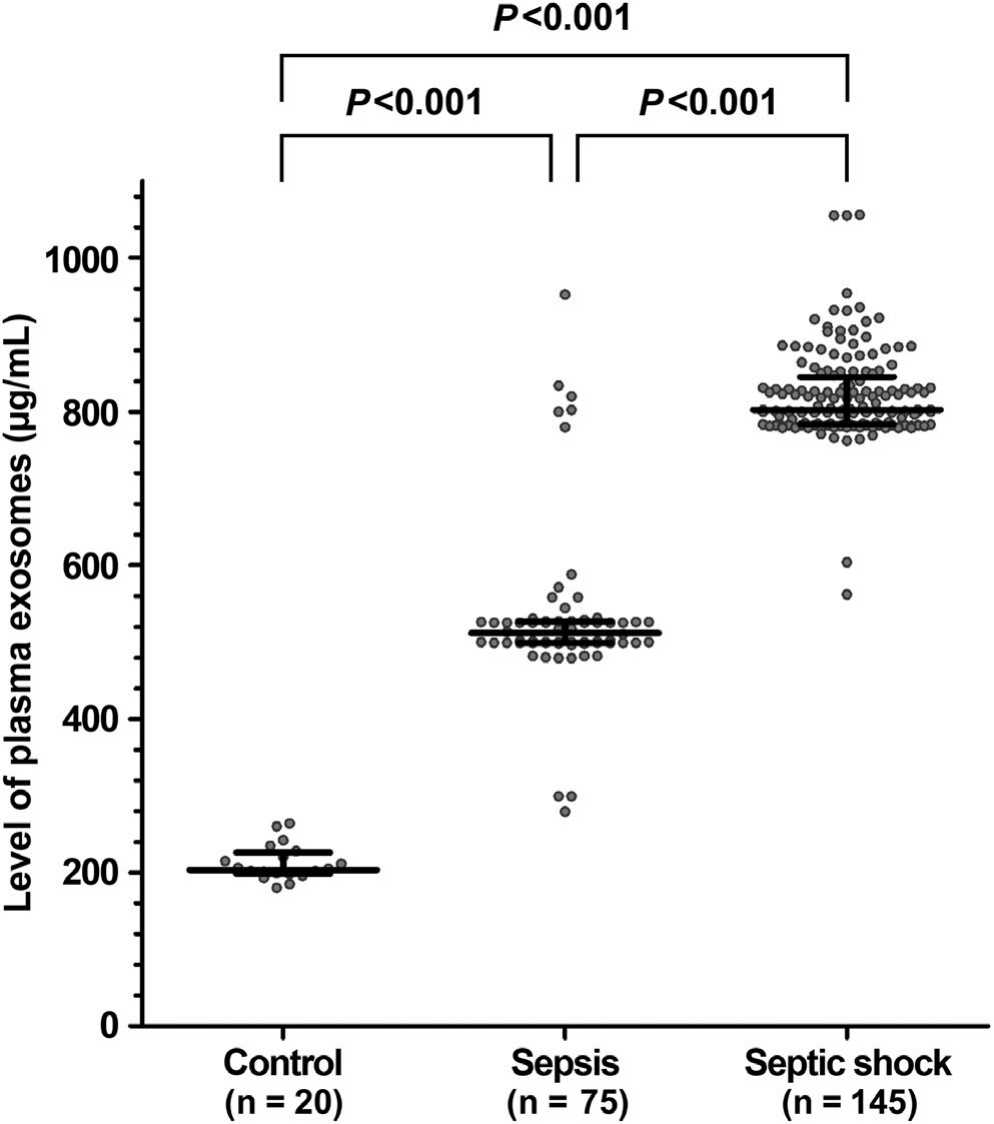
Plasma exosome levels in control, sepsis and septic shock groups. The line in the middle indicates median, and lines in the top and bottom indicate interquartile ranges of plasma exosomes

Exosome was measured in all patients. The median exosome levels were 795 µg/mL (558‐826 µg/mL). Patients with septic shock had higher plasma exosome levels (802 µg/mL, range 783‐839 µg/mL) compared with healthy controls (204 µg/mL, range 199‐222 µg/mL) and patients without septic shock (525 µg/mL, range 499‐575 µg/mL; Figure 1, *P* < .001).

### 3.2 Plasma exosome levels as a prognostic predictor of mortality and illness severity

**FIGURE 2:**
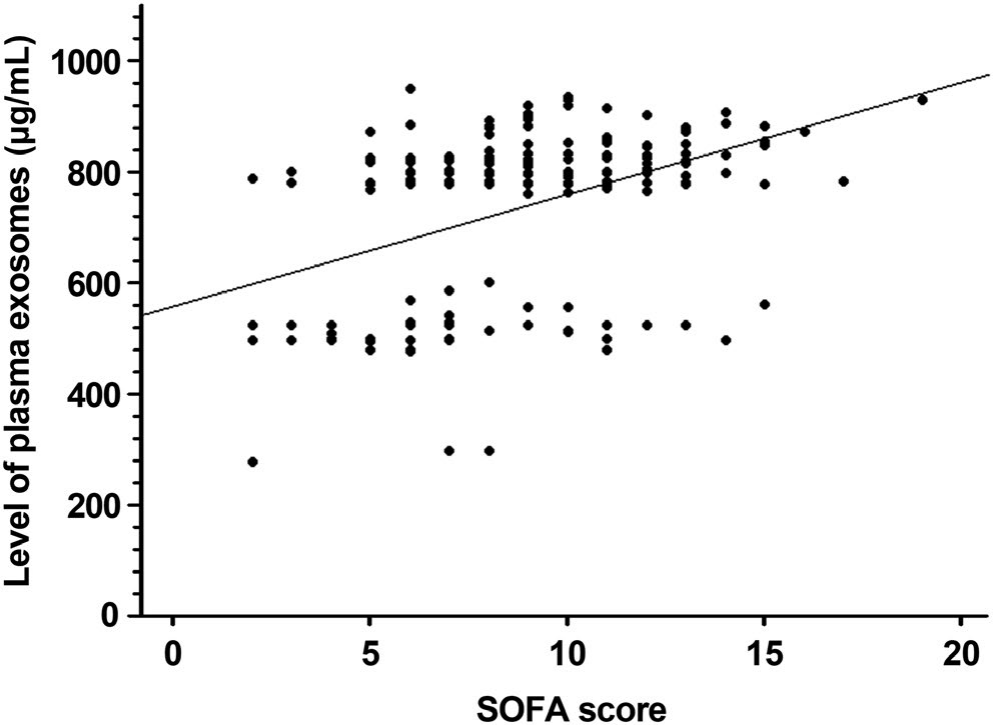
Correlation between plasma exosome levels and Sequential Organ Failure Assessment (SOFA) score in patients with sepsis. Slope: 20.2 (95% CI; 15.1‐25.4), *r* ^2^: 0.21, Pearson's *r*: 0.47 (*P* < 0.001)

**FIGURE 3:**
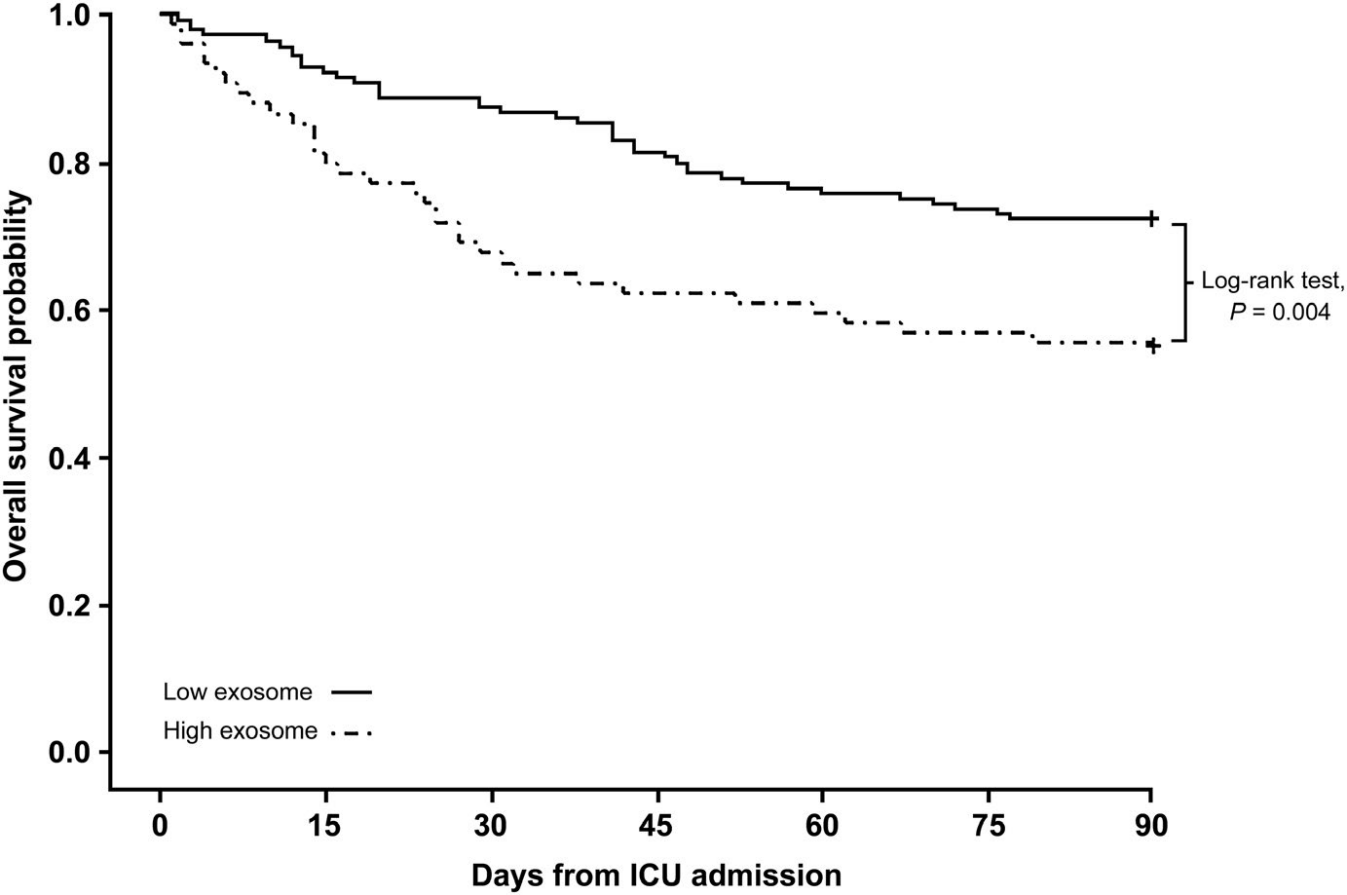
Kaplan‐Meier survival estimation of patients with high and low plasma exosome level (log‐rank *P* = 0.004)

The association between plasma exosome levels and severity of organ failure was assessed by linear regression method. There was a positive linear relationship between plasma exosome levels and SOFA scores in the study cohorts (*r* value = 0.47; 95% CI 0.36‐0.56) (Figure 2). To analyse the relationship between plasma exosome level and 28‐day mortality in the study cohort, the optimal cut‐off value of plasma exosomes was calculated using Youden's index. The best cut‐off point was 809 μg/mL in the relationship between plasma exosome level and 28‐day mortality. Patients were reclassified into two groups of high and low exosome levels based on the optimal cut‐off level, and initial diagnosis, clinical status, severity of illness and 28‐day mortality were compared between the groups. The group with exosome levels higher than 809 μg/mL was significantly associated with septic shock, need for mechanical ventilation or vasopressor support, severity of illness defined by SAPS 3, APACHE II and SOFA scores, and 28‐day mortality (Table 2). Furthermore, Kaplan‐Meier survival estimation demonstrated a significant difference in 90‐day survival between patients with high and low plasma exosome level (log‐rank *P* = 0.004; hazard ratio for death 1.92; 95% CI, 1.21‐3.05) (Figure 3).

**TABLE 2:** Outcomes and illness severity among patients dichotomized by exosome level ≥809 and <809 µg/mL

	Low exosomes (n = 146)	High exosomes (n = 74)	*P*‐value
Diagnosis			
Sepsis	69 (47)	6 (8)	<.001
Septic shock	77 (53)	68 (92)	
Clinical status on ICU admission			
Need for mechanical ventilation	53 (37)	38 (52)	.030
Need for vasopressor support	94 (64)	72 (97)	<.001
Severity of illness			
SAPS 3 score	52 (44‐60)	58 (52‐69)	<.001
APACHE II score	24 (18‐29)	25 (21‐31)	.036
Initial SOFA score	7 (6‐10)	10 (8‐13)	<.001
Mortality			
28‐d mortality	16 (11)	23 (31)	<.001
In‐hospital mortality	26 (18)	27 (37)	.004
90‐d mortality	43 (27)	33 (45)	.010

Note

Data are presented as median (interquartile range) or number (%).

Abbreviations: APACHE, Acute Physiology and Chronic Health Evaluation; ICU, intensive care unit; SAPS, Simplified Acute Physiology Score; SOFA, Sequential Organ Failure Assessment.

## 4 DISCUSSION

In the present study, the role of plasma exosome level as a potential diagnostic marker for septic shock was assessed and whether plasma exosomes could help predict the prognosis of patients with sepsis was evaluated. The result of our study indicated that the plasma exosome level was significantly higher in septic shock patients than that in sepsis patients and healthy volunteers. In addition, plasma exosome level was associated with severity of organ failure and predictive of mortality.

To the best of our knowledge, this is the first study to comprehensively evaluate the association between plasma exosome levels and severity and mortality of critically ill patients with sepsis or septic shock. The strength of our study lies in the quantification of overall exosomes in a relatively larger cohort of critically ill patients, whereas most of the preliminary studies were conducted in either animal models or focused on characterization of specific exosome contents or in small number of cohorts. As was evident in previous studies, exosomal contents such as miRNA or proteins from diverse origins provoke or prevent myocardial dysfunction, acute lung injury and acute kidney injury.^14^, ^27^, ^28^, ^29^ A murine model showed exosomal microRNA (miR‐126) derived from endothelial progenitor cells was associated with microvascular dysfunction^28^; however, the role of exosomal microRNA was not confirmed in humans. In addition, the specific plasma exosomal protein associated with progression of sepsis has been identified in sepsis patients,^30^ but cannot be generalized to date. In another study, differences in exosomal microRNA expression in healthy volunteers, patients with sepsis and patients with septic shock were established using next‐generation sequencing and quantitative reverse transcription‐polymerase chain reaction.^31^ Although three microRNAs were associated with mortality, the extraction and analysis method for microRNAs may have been too complicated for use as a routine biomarker, especially for critically ill patients who require rapid laboratory results. Finally, Real et al^32^ recently reported that expression patterns of exosomal miRNAs from sepsis patients were related to clinical outcomes based on clustering analysis. Nonetheless, the number of included patients was relatively small and only clustering pattern analysis was performed which cannot be clinically applied. Based on previous results, overall levels of plasma exosome and exosomal contents could be reasonable data points for assessing severity or mortality of sepsis. However, no studies have yet proposed the association of plasma exosomes with severity of organ failure and mortality in patients with sepsis. In this context, our data clearly showed that the overall levels of plasma exosome in sepsis patients were associated with organ failure and mortality. As there is no ideal biomarker verified for sepsis, combination of severity illness score, plasma exosome and other biomarkers should be validated for their prognostic power in predicting severity and mortality.

Compared to previous studies, our data clearly showed that the overall level of plasma exosome in sepsis patients was associated with organ failure and mortality, suggesting its potential as a biomarker for assessing severity and predicting mortality of sepsis. However, additional multicenter studies are required to confirm its efficacy and reliability as a biomarker for sepsis. Furthermore, considering the fact that there is no validated single marker for diagnosis and prognostication of sepsis, it seems necessary for the future investigations to evaluate the competency of exosome not only as a single biomarker compared to the known markers or scoring systems but also the efficacy of combining exosome with other biomarkers or incorporating it into existing scoring systems.

Of note, pan‐exosome antibody of CD9 was used in our study to capture exosomes in whole, therefore, not specific contents of exosomes such as protein, lipid, mRNA or miRNA but plasma level of overall exosome was measured. Most of the earlier studies focused on particular exosomal constituents and their role in pathogenesis and outcome of sepsis as in the cases of miR‐126 or miR‐223.^28^, ^33^ However, our knowledge on the function of exosomes is still limited to target a specific exosomal component and interpret its association with sepsis. Furthermore, exosomes possess the ability as a cell‐to‐cell communicator, triggering changes in broad spectrum of physiological and pathological processes in the acceptor cells. Previous studies demonstrated that exosomes are implicated in the development of organ dysfunction through cell‐to‐cell communication or organ crosstalk in sepsis.^34^, ^35^, ^36^, ^37^ This finding suggests that exosomes may play a role in organ failure and sepsis without evident changes in the level of specific contents. In this regard, we tried to analyse the relationship between overall exosome and severity and mortality of sepsis. However, further studies are warranted to determine the role of specific exosomal contents as well as mechanism of action of exosomes in sepsis.

The present study had several limitations. First, the study was conducted at a single referral centre, which may limit the generalizability of the data. Second, subsets of exosomal miRNAs and proteins were not analysed as potential biomarkers in the study. Previous studies have illustrated that various exosomal contents influence the pathogenesis of sepsis and host response in diverse ways. Although overall quantitation of exosome may be an advantage and point of differentiation in our study, further studies including subset analysis of exosomal miRNAs and proteins could strengthen the analysis and diagnostic potential of exosomes in sepsis. Finally, chronological changes in plasma level of exosomes were not measured in our study. Delayed peak expression in most of the cytokines and chemokines in exosomes compared with free cytokines and chemokines has been observed in a mouse model of sepsis.^15^ Serial measurement of plasma exosome level may offer more insight into understanding the kinetics of exosomes and interpreting its role as a diagnostic and prognostic biomarker in sepsis.

## 5 CONCLUSIONS

In conclusion, the study results indicate that plasma exosome levels were associated with severity of organ failure and predictive mortality in critically ill patients with sepsis.

## CONFLICTS OF INTEREST

The authors disclose that they do not have any conflicts of interest.

## AUTHOR CONTRIBUTION

**Yunjoo Im:** Data curation (equal); formal analysis (equal); investigation (equal); methodology (equal); writing – original draft (equal); writing – review & editing (equal). **Hongseok Yoo:** Data curation (equal); formal analysis (equal); investigation (equal); methodology (equal); writing – original draft (equal); writing – review & editing (equal). **Jin Young Lee:** Data curation (equal); investigation (lead); writing – review & editing (equal). **Junseon Park:** Data curation (equal); investigation (equal); writing – review & editing (equal). **Gee Young Suh:** Investigation (equal); writing – review & editing (equal). **Kyeongman Jeon:** Conceptualization (lead); data curation (equal); formal analysis (equal); funding acquisition (lead); investigation (equal); methodology (equal); project administration (lead); supervision (lead); writing – original draft (equal); writing – review & editing (lead).

## ETHICS APPROVAL AND CONSENT TO PARTICIPATE

The study was approved by the institutional review board of Samsung Medical Center (IRB no. 2013‐12‐033). Written informed consent was obtained from patients or their legally authorized representative prior to enrolment.

## Supporting information

Fig S1‐S2

## Data Availability

The data set used in this study is available from the corresponding author on reasonable request.
