# Supplementary material for: Association of plasma exosomes with severity of organ failure and mortality in patients with sepsis
Source: J Cell Mol Med. 2020 Jul 8;24(16):9439–45. doi: 10.1111/jcmm.15606 (PMC7417686; doi:10.1111/jcmm.15606)

**Online supplement**

**Association of plasma exosomes with severity of organ failure and mortality in patients with sepsis**

Yunjoo Im, Hongseok Yoo, Jin Young Lee, Junseon Park, Gee Young Suh, Kyeongman Jeon

**Supplementary Figure Legends**

**Supplementary figure S1.** Transmission electron microscopy shows vesicles with characteristic morphology and size of exosomes. Scale bar, 100 nm


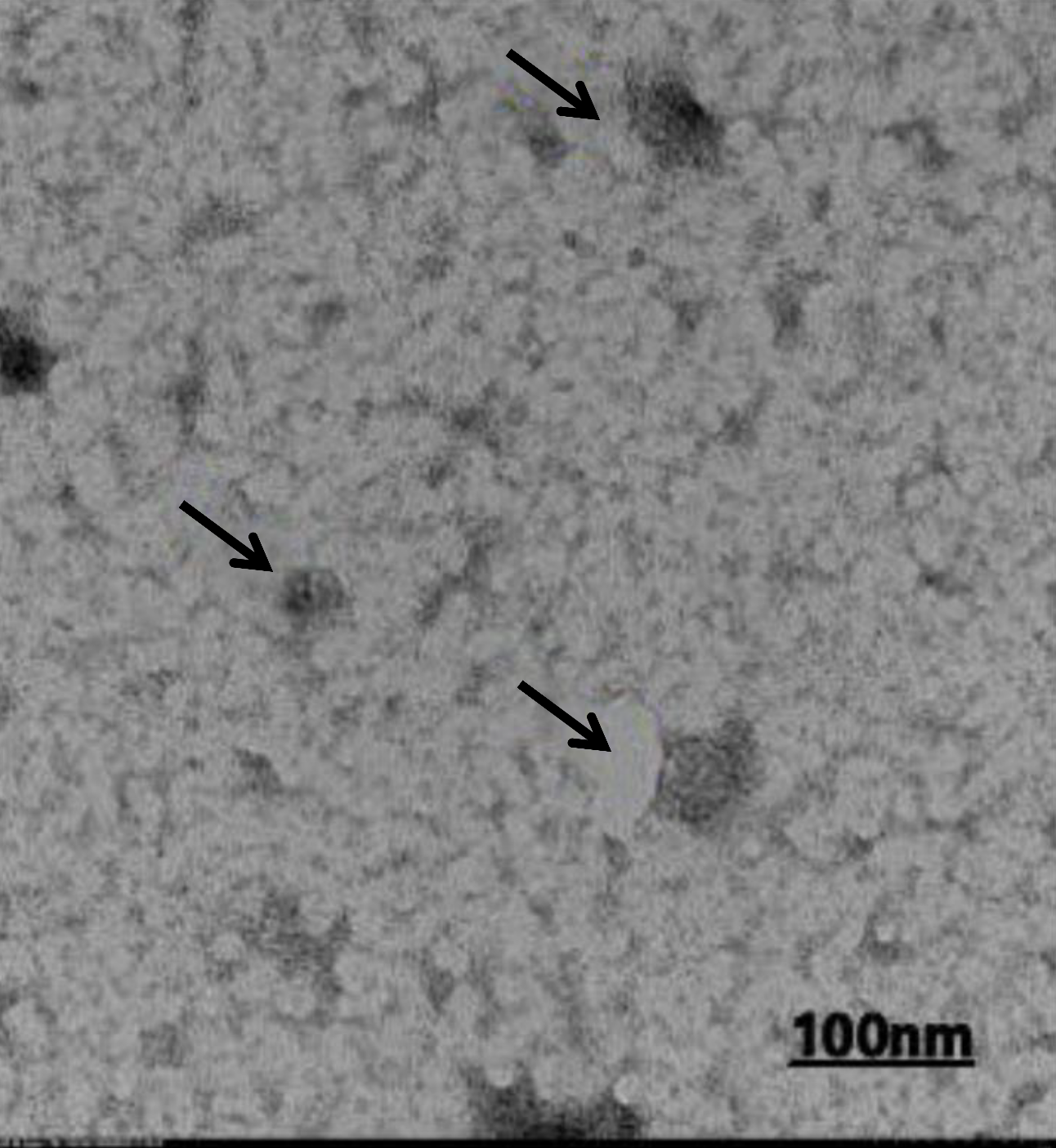


**Supplementary figure S2.** Characterization of the isolated exosomes by Western blot. Precipitation kit-purified plasma exosomes were characterized by investigating the presence of common exosome markers (CD9 and CD63) in a healthy control, two of sepsis patients and two of septic shock patients. The samples were collected in 2014 and 2019, respectively, to demonstrate the stability of the samples.


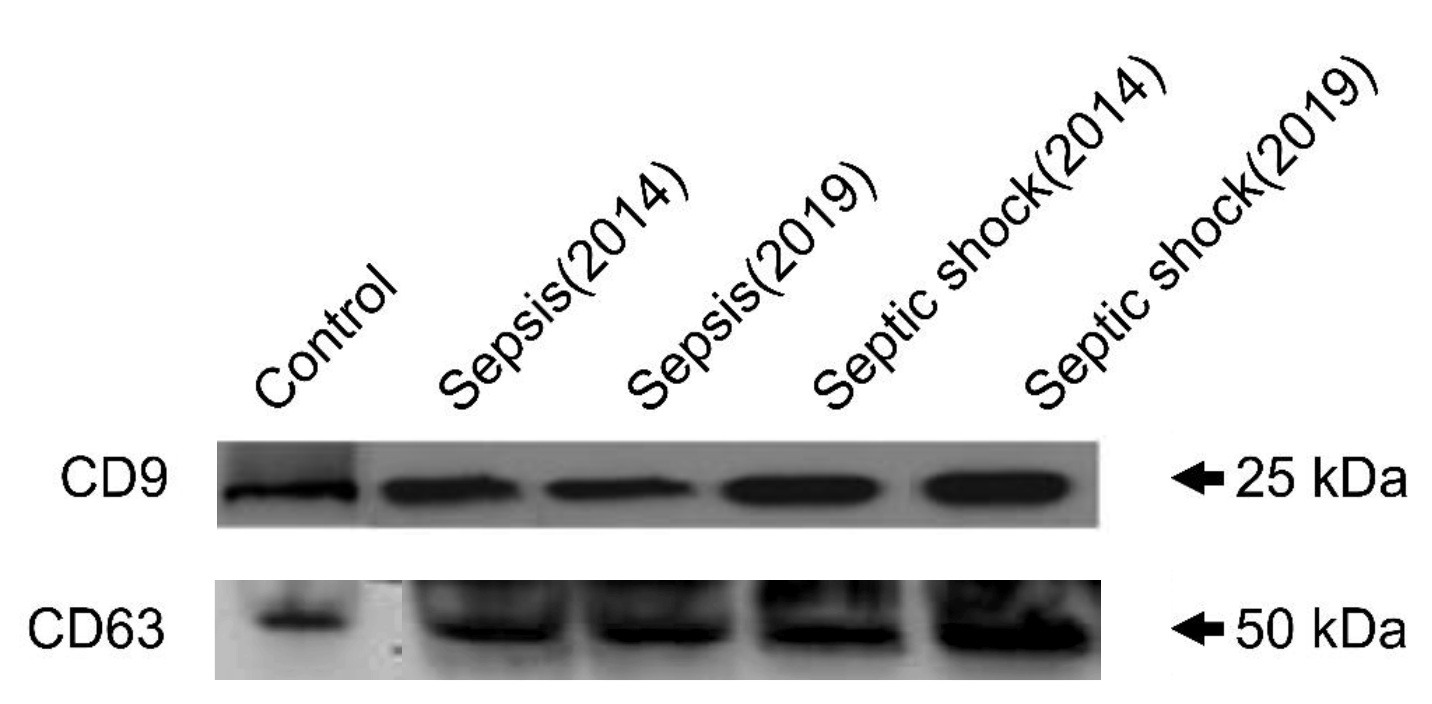

Supplement: Supplementary file 1 — Fig S1‐S2 [file JCMM-24-9439-s001.docx]
